# 

**Published:** 1859-08

**Authors:** 


### DENTAL REGISTER

### OF THE WEST.

EDITED BY

J. TAFT AND GEO. WATT.

August, 1859.

MONTHLY,

$3.00 per Year, in Advance.

CINCINNATI:
J. T. TOLAND, PUBLISHER AND PROPRIETOR.
1859.

